# The role of dairy alternatives in just food system transitions: a scoping review

**DOI:** 10.1007/s10460-024-10659-z

**Published:** 2024-11-14

**Authors:** Georgie Hurst, Laxmi Prasad Pant

**Affiliations:** Natural Resources Institute, University of Greenwich, Central Avenue, Chatham Maritime, Kent, ME4 4TB UK

**Keywords:** Just transition, Dairy alternatives, Food systems, Plant-based milks, Food justice, Dietary transition

## Abstract

Alternatives to dairy products are becoming part of mainstream food culture in Western societies amidst growing concerns for more sustainable and healthy diets, and improved animal welfare. As more citizens opt for plant-based milks, markets have responded with an abundance of alternatives to facilitate the transition. Previous systematic reviews have examined the environmental and health impacts of plant-based products compared with dairy, yet a synthesis of empirical research on the social and environmental justice impacts of dairy alternatives is lacking. This systematic scoping review aims to explore the extent, range, and nature of peer-reviewed studies on dairy alternatives that address their outcomes for justice. Publications were sought through systematic search of two comprehensive electronic databases (Scopus and Web of Science) and screened by two independent reviewers following eligibility criteria. Included studies predominantly focused on consumer identities and values, and the market-led nature of dairy alternatives consumption. Gaps in the literature included impacts on nutritional equity and the innovation and adaptive capabilities of food producers and workers across geographies. We conclude that the heavy-handed regulation of dairy alternative terminology and the market-driven nature of this transition raise questions about injustices in governance and innovation in this sector.

**Supplementary Information:**

The online version contains supplementary material available at 10.1007/s10460-024-10659-z.

## Background

The production and consumption of alternatives to dairy products, such as plant-based milks (PBM), have rapidly increased in the last decade, particularly in high-income countries (Mylan et al.^2019^). These trends are concomitant with widespread calls from scientists, including the International Panel on Climate Change (IPCC) and Lancet Health, for a transition away from animal-sourced foods and towards plant-based alternatives for more healthy and sustainable diets, reductions in greenhouse gas (GHG) emissions, and improved animal welfare (Shukla et al. 2019; Willett 2019). Industrial dairy production is associated with excessive land and water use, animal cruelty, eutrophication, and GHG emissions (Poore and Nemecek 2018; Clay et al. 2020a). Meanwhile, companies innovating in the dairy alternatives sector position their products as meeting consumers’ growing concerns for the environment, health, and animal welfare through strategic branding, labelling and marketing of alternatives as more ‘wholesome’ foods (Clay. et al. 2020b, p. 11 ). Recent innovations in the dairy alternative sector also include the emerging categories of cell-based and fermentation-derived dairy, made using advanced bioengineering processes (Mendly-Zambo et al. 2021). Although these alternatives are promising, they still face technical challenges in scaling and are yet to gain regulatory approval (Mendly-Zambo et al. 2021). Trends indicate that PBM, the largest dairy alternative category, are beginning to attenuate the dominance of fluid dairy: between 2020 and 2022, European unit sales of PBM grew by 20% while conventional milk unit sales decreased by 9%, indicating that a shift away from fluid dairy is potentially underway (GFI 2023; Clay et al. 2020b).

With the food system accounting for one-third of global GHG emissions (Crippa et al. 2021), and animal-sourced foods constituting 57% of all emissions from food production, a dietary transition away from meat and dairy products is considered necessary to achieve net zero emissions in agri-food systems (Xu et al. 2021; Kaljonen et al. 2021; Nevalainen et al. 2023; Dagevos 2021). Meanwhile, the current global food system is not ensuring good nutrition for all, as the triple burden of malnutrition (undernutrition, micronutrient deficiencies, and overweight and obesity) affects approximately 3 billion people worldwide, while worsening inequalities exacerbate food insecurity (FAO 2009; HLPE 2023). Considering the urgent need for action across multiple indicators, researchers are increasingly calling for an assessment of the risks and opportunities to justice and equality in different food system sustainability pathways, to support co-benefits and mitigate trade-offs to different human and non-human actors across dimensions, scales and geographies (Blattner 2020; Kaljonen et al. 2021; Valsta et al. 2022; Bos et al. 2018). As a result, just transition frameworks and approaches have seen burgeoning attention in scholarship on sustainable food systems transitions and transformations (Kaljonen et al. 2021; Tribaldos and Kortetmäki 2022).

The just transition was first conceptualised by labour unions to address the social consequences of environmental protection policies, and secure rights for workers in the transition away from polluting industries (Wang and Lo 2021). The concept has since evolved into a framework within the sustainability transition and energy justice literature, assessing the broader socio-economic risks associated with decarbonisation in the energy sector and developing recommendations for more equitable transition governance (Evans and Phelan 2016). To evaluate and secure justice in sustainability transitions, just transition research and frameworks predominantly make use of the three major dimensions of justice established by political theorists: distributive, procedural, and recognitional justice (Rawls 1971; Sen 1993; Schlosberg 2007; Fraser 2009; Young 1990). Distributive justice refers to the fair and equal distribution of benefits and burdens across society, with a focus on economic, environmental, and material resources (McCauley and Heffron 2018). Distributive concerns primarily imply the liberal idea of justice as fairness (Rawls 1971), though just transition and environmental justice scholars have advocated adoption of Nussbaum (2011) and Sen’s (1993) capabilities approach to the conceptualisation of distributive justice, which argues that fair distributions of material resources must also be accompanied by dignity, agency, self-determination and wellbeing (Schlosberg 2013; Edwards et al. 2016; McCauley and Heffron 2018; Kaljonen et al. 2021; Huttunen et al. 2022). Building upon Rawls (1971) and the distributional paradigm, Sen (2009) questions whether legitimate institutions and formal rules are enough, in favour of his idea that realisation of justice is only possible through capability building, empowerment and positive freedom. However, the liberal idea of justice does not satisfactorily differentiate legitimacy and justice– while the evaluative criteria of the former involve democratic processes, that of the latter entails moral values and principles (Hinsch 2010). 

The emphasis on the need for legitimate institutions and rules to ensure the realisation of justice forms the basis for the inclusion of procedural justice as another key justice dimension. Procedural justice refers to fair processes, participative decision-making, and enhanced capabilities (including epistemic qualities of knowledge and skills) (Young 1990; Fraser 1997; Schlosberg 2007; Dieleman 2015). Responsible innovation, which is of significance to sustainability transitions reliant on novel technologies, also advocates for anticipatory governance as a procedural requirement. This approach entails anticipation of risks, consequences, and impacts, reflexivity on values and beliefs, inclusive co-design processes, and responsiveness to emerging challenges and contexts (Stilgoe et al. 2013; Sovacool et al. 2021). Finding that misrecognition of marginalised social and cultural identities often forms the basis for maldistribution and political exclusion, Young (1990) advocates the inclusion of recognitional justice as a foundational principle for procedural justice, as it entails the rights, respect for, and dignities of different social groups, identities, human values, cultural traditions, knowledge, and perspectives (Fraser 2008). Together, distributional, procedural and recognitional justice have become essential to the study of just processes and outcomes within sustainability transitions and have been used as a guiding framework across both just transition research, frameworks and policy (Wang and Lo 2021).

Just transition is becoming increasingly relevant to achieving the objectives of a sustainable, inclusive, and healthy food systems transformation (Tribaldos and Kortetmäki 2022). The potential conflict between environmental sustainability, animal welfare, public health, and socio-economic wellbeing is a key tension in food systems transitions with implications for justice across multiple dimensions (Valsta et al. 2022; Bos et al. 2018; Morris et al. 2021). As a result, scholarly engagement with justice conceptualisations in sustainable food systems transition research is growing, bringing additional contributions from food justice and multi-species justice to adapt the criteria to food-related contexts (Kaljonen et al. 2021; Huttunen et al. 2022; Tribaldos and Kortetmäki 2022; Celermajer et al. 2021). Insights from food justice have uncovered the unequal distributions of power, income, nutrition, health and wellbeing that characterise the global food system and industrial food regimes (Gottlieb and Joshi 2010; Clapp et al. 2018; and Coulson and Milbourne 2021). Scholars have brought attention to labour rights and the poor conditions of those working in the food system (Lo and Jacobsen 2011), as well as the barriers low-income and minority communities face in accessing and participating in the movement towards more healthy and sustainable diets (Alkon and Agyeman 2011). These perspectives highlight the need for an emphasis on justice in sustainable food system transitions, and to question the reliance upon responsible market-based solutions that have no political obligation to address existing or potential inequalities (Agyeman and McEntee 2014). Non-human animals and ecosystems are also fundamental subjects of injustice in food systems, but they have long been invisible in justice theorising (Celermajer et al. 2021; Verkuijl et al. 2023). A just and sustainable transformation of the food system necessitates the recognition of non-human animals and nature, especially as low carbon practises may conflict with the welfare of animals or biodiversity (Kaljonen et al. 2021; Tribaldos and Kortetmäki 2022; Shields and Orme-Evans 2015).

Dairy and its alternatives exemplify these key tensions in food systems transitions. Dairy production is widely heterogenous, though significant intensification and consolidation in the recent decades has ushered in a host of socio-ecological implications, including an erosion to dairy cow welfare, the decimation of pasture-based and small-scale farming systems, significant environmental pollution and excessive resource use (Clay et al. 2020a; Bojovic and McGregor 2023). Deregulation in major temperate dairy producing regions of North America, the European Union, Australia, and New Zealand has driven such intensification and increased the vulnerabilities of smaller farms (Clay et al. 2020a). To move away from the injustices of current dairy production systems, it is imperative to interrogate both the promises and pitfalls of dairy alternatives as a more just and sustainable solution. Currently, reviews covering dairy alternatives focus only on new and emerging technological advancements (Sethi et al. 2016; Vallath et al. 2022; McClements et al. 2019; Silva et al. 2020), nutritional differences (Berardy et al. 2022; Paul et al. 2020; Vallath et al. 2022; Silva et al. 2020) and environmental footprint comparisons (Berardy et al. 2022; Carlsson Kanyama et al. 2021). One previous review has investigated emergent social science research themes on meat and milk alternatives in the protein transition (Lonkila and Kaljonen 2021). However, a synthesis of the evidence on dairy alternatives in a just food system transition is lacking, therefore an assessment of current research is necessary. This review aims to contribute to an emerging body of literature examining just sustainability transitions in food systems (Kaljonen et al. 2021; Huan-Niemi et al. 2020) through scoping evidence that addresses justice dimensions in relation to dairy alternatives. Dairy alternatives, also known as non-dairy substitutes or plant-based milks, are products designed to replace milk and other dairy products derived from animal sources. We include products made from various plant sources such as nuts, seeds, grains and legumes, as well as novel and emerging products in the cell-based and fermentation-derived categories. Despite limited research in the domain of fermentation and cell-derived dairy, the authors chose to incorporate these in the search terms as they are highly relevant to future implications for the dairy sector.

As dairy alternatives are infrequently framed in terms of their wider societal impacts, this scoping review synthesises the evidence on dairy alternatives from social, economic, cultural, and political perspectives, and analyses these results according to their study characteristics, research focus, themes and the risks or benefits to different dimensions of justice they address in the food system. This study highlights gaps in knowledge regarding the potentials and pitfalls of dairy alternatives for social and environmental justice in food system transitions, and recommends areas for future research.

## Methods

Systematic scoping reviews address broad research questions and can be used to better understand the content and extent of current literature on a topic (such as main concepts, theories, types of data and populations, and methods used). Therefore, this review addresses the following research question: what is the evidence on justice issues associated with dairy alternatives? This review employs the guidelines of the Preferred Reporting Items for Systematic Reviews and Meta-Analyses extension for scoping reviews (PRISMA-ScR) (Arksey and O’Malley 2005; Levac et al. 2010; Tricco et al. 2018). The methods to be conducted follow the nine steps proposed by the JBI scoping review framework (Peters et al. 2015): define review objectives and questions; develop inclusion criteria; describe the planned approach to searching, selecting, extracting and charting evidence; search for evidence; select sources of evidence (with title, abstract, and full-text screening); extract the evidence; chart the evidence; synthesis and summary of results; and consultation exercise. As part of consultation, this research was presented at a showcase for associate partners of the UK Food Systems– Centre for Doctoral Training.

### Search strategy

To identify relevant studies, one researcher searched using databases Scopus and Web of Science, using key terms related to the objectives of this review. These sources were chosen since Scopus and Web of Science are the two largest bibliographic databases covering peer-reviewed articles from almost all disciplines (Chadegani et al. 2013).

Since the objective of this review was to scope the evidence on the role of dairy alternatives in just food system transitions, search terms were created based on the terminology surrounding different justice dimensions and dairy alternatives, as shown in Table 1. Boolean operator OR was used to combine individual keywords for either justice or dairy alternative terms. The Boolean operator AND was used to combine key words for justice dimensions with key words for dairy alternatives. This scoping review assesses both a nascent product category and emerging research field of justice in food system transitions, therefore it was important to make use of literature to identify keywords, since terminology relating to both dairy alternatives and justice dimensions is ambiguous and not clearly defined (Lonkila and Kaljonen 2021). For dairy alternative terms, we made use of Lonkila and Kaljonen’s (2021) review of meat and milk alternatives to identify the most salient keywords, which led to the inclusion of terms for specific plants or pulses to achieve higher sensitivity of searches and comprehensive search results. We also included search terms for cell-cultured milks, and subsequently expanded our search to include the terms “fermentation-derived dairy” and “yeast-derived dairy” based on a reviewer’s recommendation. Since Tribaldos and Kortetmäki (2022) and Kaljonen et al. (2021) map and develop system-specific criteria, principles, and questions relevant to just food system transitions, we selected our justice search terms based on the most salient keywords relevant to each justice dimension between these papers. As a result of the broad nature of this search, the selection stage was essential to finding and including relevant studies. The two authors independently screened studies according to eligibility criteria (below) first by title and abstract, and then full text and conflicts were resolved in a series of meetings.

**Table 1:** Search terms

Dimension	Key words
Distributional justice	“livelihood*” OR “farmer” OR “producer” OR “social” OR “economic” OR “socio-economic” OR “access*” OR “afford*” OR “cost*” “food security” OR “equity” OR “politic*” OR “resource*” OR “inequal*” OR “labour” OR “food system” OR
Procedural justice	“democra*” OR “participat*” OR “govern*” OR “power” OR “engage*” OR “policy” OR “decision*” OR “decision-making” OR “politic*”OR “regulat*” OR “depoliticis*” OR “market-led” OR
Recognitional justice	“lifeways” OR “identit*” OR “values” OR “narratives” OR “cultur*” OR “knowledge” OR “agency” OR “esteem” OR “diversity” OR “perspective*” OR “ethic*” OR “wellbeing” OR “activist” OR “consumer” OR “perceptions” OR “awareness” OR “market*” OR “media” AND
Dairy alternatives	“plant-based milk” OR “plant milks” OR “plant based milk” OR “non dairy milk” OR “cell-cultured milk” OR “cellular agriculture milk” OR “fermentation-derived dairy” OR “yeast-derived dairy” OR “dairy free milk” OR “dairy alternative” OR “soy milk” OR “oat milk” OR “almond milk” OR “coconut milk” OR “rice milk”

### Eligibility criteria

Relevant studies for the review were chosen if they followed several inclusion criteria. We selected for peer reviewed, English language, original and full-length empirical research which focused on plant-based, cell-based, yeast-based and fermentation-derived dairy alternatives, published from 2010 to 2023. This date range was chosen since 2010 was the year that the UN Environment Program published its landmark report calling diets heavy in meat and dairy ‘unsustainable’ (Hertwich 2010); the decade that followed has also been regarded as the era when vegan and plant-based diets went ‘mainstream’ (Hancox 2018). Papers using qualitative, quantitative, or mixed methods were included, though they had to have some relevance to social and environmental justice research domains. We sought to include papers that clearly presented either challenges or opportunities associated with dairy alternatives (these were also identified by any synonyms e.g., “risk” or “benefit”), and that had relevance to justice dimensions of distribution, recognition, or procedure. To support both the selection and data analysis stage, a sample of research questions relevant to the role of dairy alternatives in just food system transitions were developed, and are presented in Table 2. These questions are based on Kaljonen et al.’s (2021) justice questions and tensions raised by dietary transitions, as well as Tribaldos and Kortetmäki’s (2022) criteria for just food system transitions. Additionally, we made use of Verkuijl et al.’s (2022) mapping of stakeholders and principles for a just transition in the meat sector to adapt these questions more closely to the case of dairy and its alternatives.

**Table 2:** Justice research questions for dairy alternatives

Dimension	Research questions
Distributive	What are the nutritional impacts of dairy alternatives across various socio-economic groups, and various regions or countries? How do dairy alternatives affect different socio-economic groups in terms of access and affordability? What are the effects of the dairy alternative sector on the livelihoods of farmers and other food chain workers between regions and production sectors? How is the dairy alternative sector affecting innovation capacity and value creation across food chain actors and activities? What are the path-dependencies and lock-ins that affect the adaptive capacities of different food system actors to meet new challenges of dairy alternative production? How can capabilities of farmers and other workers in food chain be developed to meet challenges of dairy alternatives? How do dairy alternatives affect the protection and welfare of non-human species and ecosystems?
Procedural	How is procedural justice affected by the current governance and facilitation of dairy alternatives? How does the governance and regulation of dairy alternatives affect existing dynamics of power and ownership in the food system? How is access to reliable information on health, nutrition and environmental impacts of dairy alternatives made available to support decision-making?
Recognitional	How do dairy alternatives support or challenge present lifeways and values associated with eating well? How do dairy alternatives challenge or support the knowledge and food literacy of diverse population groups? How do dairy alternatives affect the ethical recognition of non-human animals and the natural world?

### Data charting and analysis

One author performed the charting and analysis, and a second author validated a sample of this analysis. The data charting process was conducted using Excel with tools for charting developed for this review. The charting table included criteria chosen to allow for descriptive analysis of the characteristics of studies (e.g. discipline, year of publication, country of origin, methodology) to elucidate the scope and nature of studies (Table S1, S2). Included studies were subjected to a thematic content analysis using the software NVivo. Thematic content analysis was chosen as this method allows for theoretically driven analysis where latent justice themes can emerge from interpretation of the data (Braun and Clarke 2006).

First, texts were analysed using inductive coding to develop data-driven themes (Fereday and Muir-Cochrane 2006). Secondly, data-driven themes were categorised according to their relevance to the research questions developed for this review (Table 2). This assessment guided the classification of each theme into a corresponding theory-driven theme of distributional, procedural, and recognitional justice. Finally, the key justice themes of each text were added to the charting table, along with an analysis. To identify gaps in the literature, we utilised the research questions to evaluate how the included studies have defined the role of dairy alternatives in a just food system transition, and where this role remains unclear. We explore future research needs in the conclusion.

This review has several methodological limitations. Firstly, the field of justice is broad and spans multiple disciplines, therefore some potentially relevant terminology may have been missed from our search terms. There is also a degree of subjectivity as to what can be considered a justice implication; therefore, the selection of relevant studies reflects the reviewers’ interpretations of potential risks and benefits to justice based upon key literature in the domain of just food systems transitions. Terminological ambiguity of dairy alternatives may have also led reviewers to exclude emerging and potentially relevant key words.

## Results

**Fig. 1:**
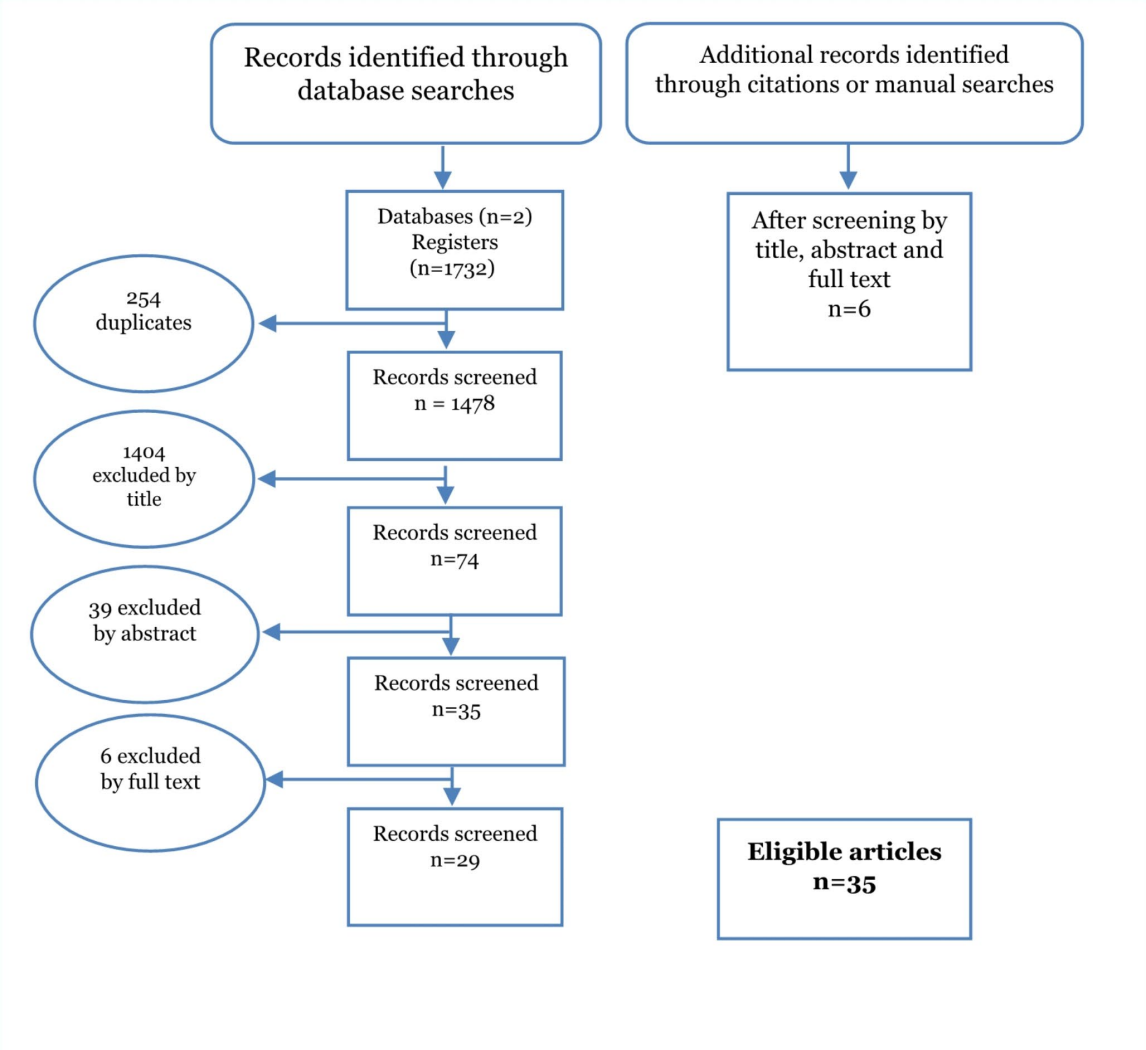
PRISMA flow chart (preferred reporting items for systematic reviews and meta-analyses) showing the selection process of eligible studies

Our search retrieved 1732 studies in the initial search, of which 35 articles met our inclusion criteria and were selected for review, including an additional six studies identified from a hand-search of bibliographies (Fig. 1). The hand-searched articles were identified in Google Scholar and institutional repositories.

### Study characteristics

Table 3 shows a descriptive summary of the included studies on dairy alternatives. Most of the included papers examined plant-based milk (*n* = 33), while only two studies focused on cell-based milks. This is likely because cell-based milks are an emerging beverage not yet on the market, so very little empirical evidence can be sought for them regarding their societal implications. The retrieved literature represented a relatively limited geographical scope, with studies spanning 10 countries across four continents. Regionally, the included studies were heavily weighted to Europe (54%) and North America (42%). This geographic scope reflects the current trend of dietary shift towards dairy alternatives, which is mostly occurring in high-income nations with historically higher consumption of animal-based foods (Lonkila and Kaljonen 2021; Clay et al. 2020b).

**Table 3:** Summary of study characteristics

Geographic region	Year of publication	Study type	Disciplines
Europe (18)— UK (6), Sweden (4), Austria (1), France (1), Italy (1), Finland (1), EU region non- specific (3) USA and Europe (1) New Zealand (1) USA (10) Canada (4) Australia (1) India (1)	2014 (2) 2017 (2) 2018 (4) 2019 (5) 2020 (7) 2021 (5) 2022 (9) 2023 (1)	Observational study (15) Case study (7) Content/discourse analysis (3) Policy and legal analyses (4) Cross-sectional study with substitution modelling (2) Econometric study (2) Experimental study (1) Theoretical study (1)	Business and marketing (13) Public health nutrition (7) Geography (5) Law and policy (4) Behavioural Economics (3) Sociology (2) Education (1)

**Fig. 2:**
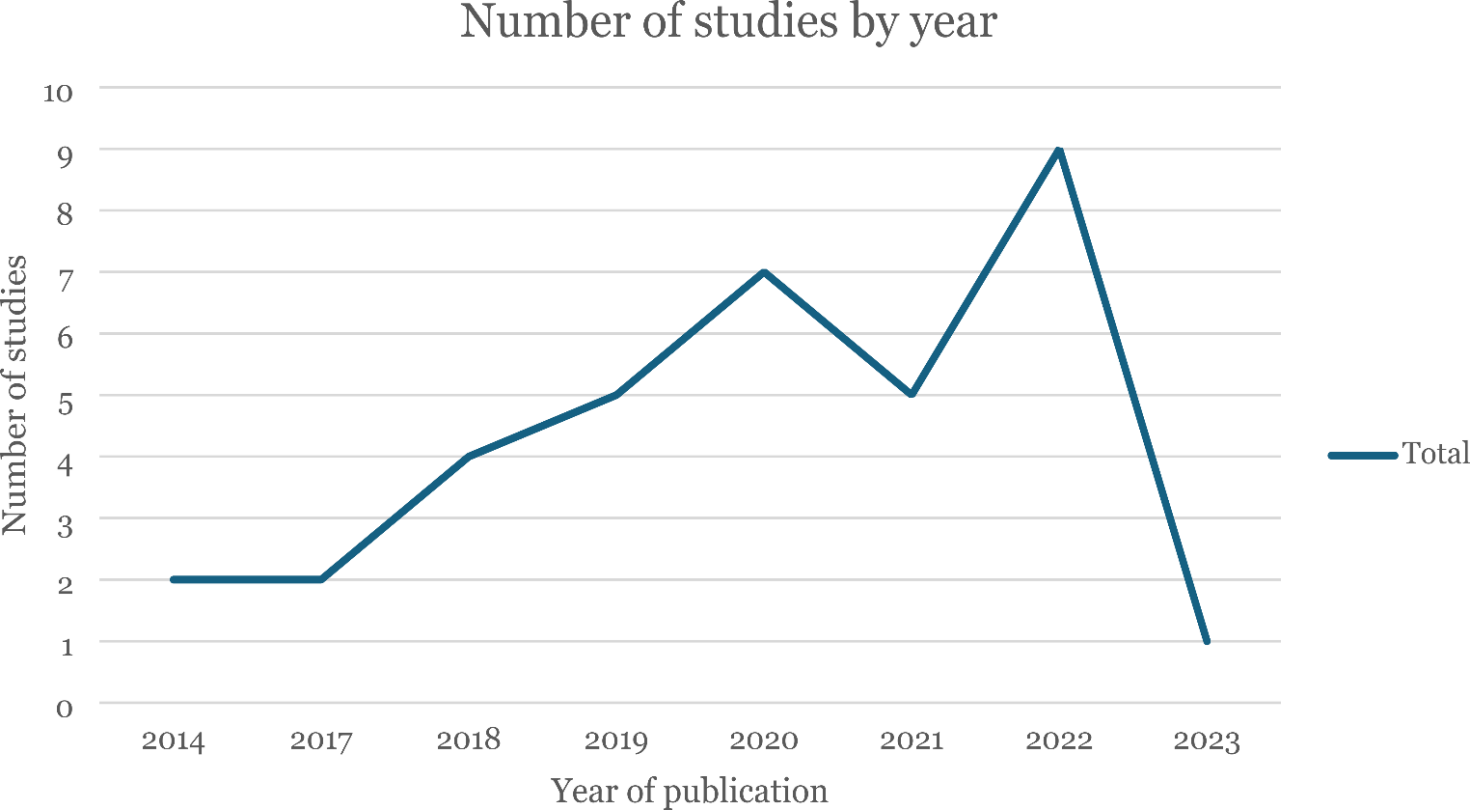
Number of included studies by year of publication

The included papers show a clear growth in the research interest on dairy alternatives over time, with no studies found before 2014, and 2022 yielding 25% of all included studies (Fig. 2). Dairy alternatives have been studied using a range of study designs, assessing challenges and opportunities from different perspectives. Most of the included literature were observational studies (*n* = 15) and case studies (*n* = 7) and within these categories were studies mainly investigating consumer acceptance (*n* = 11) and different populations’ knowledge about the nutritional or environmental impacts of PBM (*n* = 4).

Several papers (*n* = 3) employed content or discourse analyses to explore the marketing narratives and strategies of the dairy alternative sector, while some case studies (*n* = 3) also utilised content or discourse analysis as a secondary method to explore the role of marketing materials on consumer beliefs around the environmental, ethical and health benefits of dairy alternatives. Meanwhile, policy and legal analyses (*n* = 4) all explored the role of labelling regulations and public policy in supporting or hindering the establishment of dairy alternatives.

Business and marketing and public health nutrition disciplines had the highest number of publications, representing 57% of studies. This prevalence demonstrates that the research focus on dairy alternatives has mostly been consumer-oriented, with limited assessment of impacts to the supply side of the food system.

### Thematic analysis

In the first stage of thematic analysis, 9 core data driven themes were identified through NVivo coding. During the secondary stage of analysis, these themes were categorised according to the justice research question most relevant to that theme, and their respective justice dimensions. Table 4 describes the salience of the data-driven themes, their associated justice dimension, a brief explanation of the theme, and the included papers per theme. Research themes are not mutually exclusive, and the majority of reviewed papers had relevance to more than one theme and in a few cases related to more than one dimension of justice—this is reflected in the counting of themes and included papers in Table 4. However, while some papers may fall under multiple thematic categories, judging the salience of several themes per text was important for our analysis of research gaps. The secondary stage of analysis, where data-driven themes were categorised by their relevant justice dimension, revealed that concerns relevant to recognitional and procedural justice received the most attention in the literature.

**Table 4:** Salience of themes and associated justice dimensions

Justice Dimension	Data-Driven Theme	Description	Included papers
Distributive justice (*n* = 11)	Socio-economic (*n* = 6)	Affordability for consumers; risks and benefits to work and livelihoods	Zhang et al. 2020; Domke 2018; Yokessa and Marette 2019; Huang 2022; Clegg et al. 2021; Stewart et al. 2020
	Healthcare (*n* = 3)	Risks and benefits to nutrition, food security, or healthcare in different populations	Laila et al. 2021; Clegg et al. 2021; Fifi et al. 2022
	Geography (*n* = 2)	Distribution of risks and benefits across different geographies	Newman et al. 2022; Basu 2022
Procedural justice (*n* = 24)	Policy and regulation (*n* = 7)	Public policy influence on dairy alternative production and consumption	Leialohilani and Boer 2020; Domke 2018; Mikkola and Risku-Norja 2014; Huang 2022; Morris et al. 2019; Mylan et al. 2019; Leone 2019; Vallone and Lambin 2023
	Access to information (*n* = 8)	Scope and scale of reliable information for consumers to make informed decisions (e.g. regarding nutrition)	Laila et al. 2021; Morelli and Vitale 2020; Clegg et al. 2021; Jones 2022; Clark et al. 2022; Fifi et al. 2022; Wheeler et al. 2014; Vallone and Lambin 2023
	Markets and consumption (*n* = 9)	Market-led nature of dairy alternatives and ethical consumerism as a vehicle for participation in food systems	Mikkola and Risku-Norja 2014; Ledin and Machin 2020; Fuentes and Fuentes 2017; Jones 2022; Morris et al. 2019; Clay et al. 2020b; Mylan et al. 2019; Trewern et al. 2021; Mouat and Price 2018
Recognitional justice (*n* = 32)	Culture and identity (*n* = 10)	How culture, beliefs, traditions, attitudes, values, identities, and lifestyle practices affect consumption choices	Haas et al. 2019; Moss et al. 2022; Adamczyk et al. 2022; Basu 2022; Wiblom et al. 2021; Wheeler and Chapman-Novakofski 2014; Boaitey and Minegishi 2018; McCarthy et al. 2017; Schiano et al. 2022; Jaeger and Giacalone 2021
	Knowledge and awareness (*n* = 8)	Consumer food literacy and how this affects consumption choices	Allen et al. 2018; Laila et al. 2021; Adamczyk et al. 2022; Morelli and Vitale 2020; Wiblom et al. 2021; McCarthy et al. 2017 Schiano et al. 2020; Schiano et al. 2022
	Non-human animals and nature (16)	The recognition of the rights and welfare of non-human beings in dairy alternative markets and dietary choices	Morris et al. 2019; Mylan et al. 2019; Adamczyk et al. 2022; Basu 2022; Boaitey 2020; Haas et al. 2019; Laila et al. 2021; McCarthy et al. 2017; Mikkola and Risku-Norja 2014; Moss et al. 2022; Schiano et al. 2020; Schiano et al. 2022; Wiblom et al. 2021; Yokessa and Marette 2019; Mouat and Price 2018

## Discussion

### Distributive justice

Several studies were identified as addressing risks to nutritional deficiency associated with dairy alternatives, a key distributional concern of transitions away from dairy (Kaljonen et al. 2021; Valsta et al. 2022), though to varying degrees and with limited assessment across different socio-economic populations. Two studies found that variation in the nutritional quality of PBM versus dairy milk, as well as lack of fortification of some PBM products, were a risk to consumer groups where milk is a key source of necessary nutrition. In a study modelling the nutrient intake of dairy products and PBM using UK Dietary Reference Values (DRV) for each age group, Clegg et al. (2021) note substitution of dairy presents risk of nutrient deficiencies (mainly protein, calcium, iodine and B12) to infants, children, and pregnant or nursing mothers. Similarly, Zhang et al. (2020) modelled dietary scenarios in a cross-sectional study where dairy servings were substituted for plant-based alternatives, and found risk of micronutrient displacement in populations with typically suboptimal intake of key vitamins and minerals and protein. The authors also argue that dietary transition towards PBMs could impact groups where cows’ milk is a staple food for nutrition such as ‘those with restricted income and geographical access to food, such as in indigenous Australians and rural/remote residents’ (Zhang et al. 2020; p.9). There was limited evidence on dairy alternative consumption among nutritionally vulnerable population groups, such as low-income, women of reproductive age and BIPOC (Black, Indigenous, People of Colour). This evidence would support policymakers to prevent health risks, such as vitamin D deficiency among non-white populations (Cashman et al. 2016), being exacerbated by a mandated dietary transition towards dairy alternatives. With the EAT Lancet planetary health reference diet considered both unaffordable to most of the world’s population (Hirvonen et al. 2020), and containing inadequate proportions of essential micronutrients (Beal et al. 2023), nutritional equality must be a priority for just and sustainable food system transitions. Future research into enhancing the consumption of dairy alternatives should factor in critical nutrition concerns and develop considerations to mitigate potential distributional health injustices (Zhang et al. 2020; Valsta et al. 2022).

Several studies discussed socio-economic factors of dairy alternatives, though predominantly on affordability and the demand-side of the food chain (Kaljonen et al. 2021). Clegg et al. (2021) explored consequences of PBM on household expenditure and found that PBM products in the UK were almost three times the price of cows’ milk and dairy products, while higher nutrient PBMs were most expensive due to the cost of fortification, potentially exacerbating risk of micronutrient deficiencies in lower-income households substituting dairy. Findings from a comparative analysis of US and European agricultural policies indicate that higher relative cost of PBMs is due to disproportionate government spending on animal production compared with emerging alternatives, allowing dairy prices to remain low (Vallone and Lambin 2023). In an econometric study modelling the impact of a carbon tax on plant and animal-based milks on household preference, Huang et al. (2022) found that a carbon tax on dairy could ‘decrease the relative price of plant-based fresh milk products and would be likely to induce a greater shift among households towards consumption of these products’ (p. 527). Similarly, Yokessa and Marette (2019) examine strategies to reduce the consumption of dairy milk utilising a consumer ‘willingness-to-pay’ experiment, finding a mixture of taxes on regular milk and subsidies on both organic PBM and dairy maximised the consumers’ welfare and could support a decrease in GHG emissions. Both studies indicate that Net Zero policies incorporating carbon taxes on dairy could make dairy alternatives more affordable to consumers, though there is a lack of discussion regarding strategies to mitigate supply-side trade-offs of carbon taxes between small-scale free-range and large-scale intensive dairy producers and workers, rural communities in predominantly dairy producing regions, and population groups at greater risk of nutrient deficiency (Henderson et al. 2018; Valsta et al. 2022).

While Stewart et al. (2020) found that purchases of that PBM are negatively affecting those of dairy, with a rate of replacement nearing one to one in recent years, there was limited evidence of justice implications for supply side stakeholders including dairy and feed producers and workers. These gaps may reflect the lack of a discernible relationship between dairy alternatives and agricultural livelihoods currently, although these trends have implications for the long-term viability of small-scale dairy farms in an already highly consolidated sector (Clay et al. 2020a). Future studies should consider how anticipatory governance of alternatives can mitigate potential social and economic justice impacts, including farmers’ limited bargaining power against retail and industry (Muiderman et al. 2022; Stilgoe et al. 2013; Puupponen et al. 2017).

Distributional justice implications of dairy alternatives across geographies and supply chains was scarcely addressed in the literature. Basu (2022) shows that semi-urban and rural residents face barriers in the accessibility of dairy alternatives in India, presenting inequities in access to healthier, more sustainable and ethically preferable foods. On the supply side, Newman et al. (2022) conducted telecoupling analysis on prospective cell-based milk (CBM) supply chains in British Columbia (BC) in Canada, considering land use change and the biodiversity, social and economic trade-offs of a CBM transition under multiple scenarios. The study elucidated how, depending on policy direction, provincial cellular dairy transition may perpetuate cosmopolitan injustices by displacing the ecological harms of BC’s dairy industry to sugarcane producing countries (such as Brazil), due to the dependence on sugar crops in cellular dairy manufacturing. Socio-economic risks in BC were also anticipated due to its dependence on the dairy industry for employment, and the low willingness and capacity of dairy farmers to adopt new technologies, including the potential to diversify their operations into dairy alternatives.

The capabilities and innovative capacities of different food systems actors to transition into emerging dairy alternative markets is an underexplored consideration for just transitions and dairy alternatives (Sen 1999; Kaljonen et al. 2021). In particular, the barriers and opportunities in dairy alternative values chains and emerging technologies, such as fermentation and cell-derived dairy, for rural development and land use (Manning et al. 2023; Newton and Blaustein-Rejto 2021; Morais-da-Silva et al. 2022). As exemplified by Newman et al. (2022)’s study, the changing demand for land-based resources stimulated by dairy alternatives represents an important avenue for just food systems transition research. This includes examining whether such demand may exacerbate social and environmental injustices associated with input-intensive monocultures, particularly the welfare of humans and non-humans in predominantly low- and middle-income countries (Friis and Nielsen 2019; Verkuijl et al. 2022).

### Procedural justice

We found that themes related to procedural justice were prominent in the included studies. Four studies focused specifically on existing state interventions to regulate dairy alternatives in the USA (Leone 2019; Vallone and Lambin 2023) and Europe (Domke 2018; Leialohilani and de Boer 2020; Vallone and Lambin 2023). These studies revealed similarities in labelling regulations of PBMs between Europe and the USA, where restrictions have been placed on use of the term ‘milk’ for animals’ milk alternatives. All four studies suggested that these labelling restrictions were an effort to protect the dairy industry, as there is contested evidence on the justification that consumers are confused about the nutrition and nature of PBMs. Vallone and Lambin (2023) provided the most thorough investigation of public policy support for dairy alternatives compared with animal-derived dairy across both US and European contexts, finding that the instrumental power of the incumbent dairy sector drove governments to impede a transition away from animal products. This disproportionate influence resulted in agricultural financial support being directed almost entirely to livestock and feed producers, while creating regulatory hurdles for the commercialisation of dairy alternatives. The dairy industry’s overt political influence significantly undermines procedural justice by stifling innovation, limiting participation and representation of all stakeholders, and delaying the transformative actions necessary to achieve just and sustainable food systems, benefiting only a minority of actors.

Provision of reliable public health guidance on dairy alternatives was another focus of several studies with implications for procedural justice: specifically, the need to improve food literacy for more informed dietary decision-making (Tribaldos and Kortetmäki 2022; Vigden and Gallegos 2014). Clarke et al. (2022) found that US healthcare professionals doubt consumer knowledge about nutritional differences between dairy and PBM, with two-thirds of participants blaming poor labelling. However, other studies highlight that limited awareness of PBM nutrition was due to lack of clear public information (Wheeler et al. 2014) and a lack of physician knowledge and advice for parents (Fifi et al. 2022). Lack of trust in healthcare professionals was found to be a driving factor in consumption of PBMs among Italian youth, particularly as a strategy to treat digestive issues (Morelli and Vitale 2020). Finding a lack of public health guidance on dairy substitution and micronutrient deficiency, Clegg et al. (2021) recommend more public information to support the health of citizens choosing to consume PBMs. National dietary guidelines have been identified as an important mechanism to improve public food literacy about the risks and benefits of dairy substitution (Vallone and Lambin 2023). Evidence from a study of parents in Canada found that Health Canada’s Food Guide was a facilitator to PBM provision since it emphasises plant-based foods (Laila et al. 2021). Recommendations for dairy alternatives in the US and Europe remains scarce, while the link between animal products and environmental impacts in guidelines is entirely absent (Vallone and Labin 2023). Transparency on the health, nutrition and sustainability of food are essential for improved consumer awareness and can facilitate procedural justice in dietary transitions. More research is needed to determine the efficacy of different approaches to public health guidance on dairy alternatives.

Another major theme with implications for procedural justice was the market-driven nature of dairy alternatives consumption, particularly regarding the role of corporations, marketing and advertising tools and ethical consumption patterns. Critical discourse and framing analyses of dairy alternatives’ marketing have noted that brands mobilise the language of social movements to capture ethically conscious consumers (Clay et al. 2020b; Ledin and Machin 2020; Mylan et al. 2019) and investors of ‘disruptive’ innovations (Mouat and Prince 2018; p. 325). Clay et al. (2020b) characterise the transition as a ‘palatable disruption’ (p. 945), of the food system signifying consumption as a form of political engagement without entailing transformative actions outside of consumer capitalist norms. However, Fuentes and Fuentes (2017) dispute the claim that dairy alternatives lead inherently to an apolitical corporate co-option of alternative food movements. In an ethnographic study on PBM consumers they suggest that even highly commercialised and consolidated alternative markets can have a positive effect on public discourse and political engagement with food systems transformation (Fuentes and Fuentes 2017).

Mylan et al. (2019) argue that power asymmetries in the food system are entrenched by dairy alternatives, as incumbent actors less locked-in to the dairy regime - like retailers and food processors - have been crucial for ‘normalising’ (p. 242) PBMs for mass consumption by exploiting changing consumer preferences. Supermarkets have indeed been quick to respond to consumer demand for PBM products, as Jones (2022) found in an observational study of UK supermarkets. However, in interviews with UK supermarket representatives Trewern et al. (2021) note hesitation from retailer representatives to limit dairy sales in favour of plant-based alternatives due to challenges of market competitiveness and established relationships with meat and dairy suppliers. These findings demonstrate the procedural implications for power and decision-making capabilities across the supply chain with regards to dairy alternatives. While powerful dairy industry actors can restrict supermarkets from offering alternatives, dominant corporations (including dairy processing giant Danone) can seize control of new product markets, further consolidating power while marginalising smaller, emerging, or vulnerable actors as policy lags (Mylan et al. 2019). In a Finnish study of expert discourses on PBM and dairy (Mikkola and Risku-Norja 2014) and a UK study of dairy and PBM stakeholders (Morris et al. 2019), diverging and conflicting discursive frames around PBM consumption were found to be driving the lack of coordination between market and institutional actors, and stymying action to support a more responsible and anticipatory governance of a transition away from dairy. Both studies use this evidence to argue for a more pronounced role for public policy in anticipating and governing a dairy alternative transition, as opposed to solely protecting the interests of the dairy industry (Vallone and Lambin 2023).

Further research is needed to examine policy coherence between demand and supply side policies regarding dairy alternatives and the dairy sector’s social and environmental impacts on human and non-human species and ecosystems. This work can illuminate how more responsive policy intervention in the dietary transition can achieve co-benefits with agricultural sustainability transitions and rural development. These findings highlight that although earlier systematic reviews covering the transition from dairy to PBMs focus primarily on new and emerging technologies (Sethi et al. 2016; Vallath et al. 2022; McClements et al. 2019; Silva et al. 2020), neither these reviews nor the present review reveal concerns for responsible innovation of dairy alternatives. The evidence on procedural aspects of responsible innovation, such as anticipation of future, reflexivity, responsiveness and inclusion, was limited (Pant 2019; Stilgoe et al. 2013). Gaps in the literature addressing procedural concerns of dairy alternatives also include examinations of power relations within the dairy alternative sector and PBM value chains, as they may be perpetuating the existing inequities of commodity crop production systems between producers and seed manufacturers (Kaljonen et al. 2021).

### Recognitional justice

A significant number of papers were found to relate to recognitional justice, especially as it concerns the cultural acceptability of dairy alternatives by consumers (Adamczyk et al. 2022; Allen et al. 2018; Basu 2022; Boaitey and Minegishi 2018; Haas et al. 2019; Jaeger and Giacalone 2021; Laila et al. 2021; McCarthy et al. 2017; Morelli and Vitale; Schiano et al. 2020; Schiano et al. 2022). These studies were oriented towards understanding how dairy alternatives affect and are affected by: food literacy, visibility of sustainability and animal welfare; and culture and identity. This evidence suggests that dairy alternatives, particularly PBMs, do not significantly challenge existing socio-cultural norms around food preparation and consumption, making it a palatable transition for many population groups (Clay et al. 2020b; p. 945; Basu 2022; Moss et al. 2022; Schiano et al. 2022). However, it must be noted that research on this topic was based mostly in North America and Europe, therefore how socio-cultural and ethnic differences affect the acceptability of PBM transition in other regions is not known and is a gap for future research.

The popularity of dairy alternatives has been propelled by a growing trend towards ethical and sustainable consumption, especially in high income countries (Morris et al. 2019). Several studies found consumption of PBM potentially benefits consumer wellbeing since it affirms an “activist” identity, providing a sense of improving the state of the environment and animal welfare (Basu 2022; Boaitey and Minegishi 2018; Haas et al. 2019; McCarthy et al. 2017; Schiano et al. 2020). As previously mentioned, studies on PBM marketing tools utilise activist language since this framing resonates with the identities and values of a growing population group, facilitating the practice of what is perceived to be a more ethical and sustainable lifeway (Clay et al. 2020b; Morris et al. 2019; Ledin and Machin 2020; Fuentes and Fuentes 2017).

The potential for dairy alternatives to realise recognitional justice for animals and ecosystems was present in many included studies, especially those oriented around the narratives and frames of consumers and civil society organisations (Morris et al. 2019; Mylan et al. 2019; Adamczyk et al. 2022; Basu 2022; Boaitey 2020; Haas et al. 2019; Laila et al. 2021; McCarthy et al. 2017; Mikkola and Risku-Norja 2014; Moss et al. 2022; Schiano et al. 2020; Schiano et al. 2022; Wiblom et al. 2021; Yokessa and Marette 2019; Mouat and Price 2018). In a framing analysis of the discourses concerning dairy and its substitutes, Morris et al. (2019) identified dairy alternatives as key to promoting an end animal exploitation in the food system through the ease of substitution. However, these authors concluded that the most common framing of dairy alternatives was a ‘politically more palatable discourse’ which promoted partial substitution of dairy for improved human and environmental health without fundamentally challenging animal food production-consumption (Morris et al. 2019; p. 50). This finding was corroborated by Adamcyzk et al. (2022), where consumers’ ethical considerations for dairy cows were non-existent in the case of local and free-range sources, and significantly less than animals raised for slaughter. Furthermore, several studies noted that dairy alternative companies primarily highlight the health and sustainability benefits of their products while failing to acknowledge animals as beneficiaries of dairy substitution (Clay et al. 2020b; Mylan et al. 2019; Morris et al. 2019; Fuentes and Fuentes 2017). This erasure of animals as recipients of justice can undermine the full ethical dimensions of dairy avoidance, and potentially limits consumer awareness and engagement with the injustices dairy cows and other animals face in the food system.

A number of consumer-oriented studies examined themes of food literacy and nutrition knowledge. Food literacy serves as a bridge between recognitional and procedural justice in food systems transitions, as it empowers individuals to make more informed food choices for both their health and engagement with food system transformation (Yamashita and Robinson 2016; Vigden and Gallegos 2014; Cullen et al. 2015). Within this topic, we found that a transition to consuming dairy alternatives is driven by, among other aspects, higher level of health consciousness and nutrition knowledge (Allen et al. 2018; Laila et al. 2021; Boaitey and Minegishi 2018), as well as an alternativising of mainstream health and nutrition practices (Morelli and Vitale 2020). Certain types of knowledge also proved to be a barrier to dairy alternatives consumption, centring around resistance to food technology and food neophobia, traditional nutrition concerns, culinary practices, and support for dairy farmers livelihoods (Adamczyk et al. 2022; Allen et al. 2018; Haas et al. 2019; Jaeger and Giacalone, 2021; Laila et al. 2021; Schiano et al. 2022).

Overall, there was a dearth of findings relating to how dimensions of social difference and identities, including class, race and gender impact consumption, food literacy, and accessibility of dairy alternatives. These are key concerns for recognitional justice and essential considerations for designing sustainable dietary transitions initiatives and policies that do not potentially exclude, marginalise, or vilify certain groups, for their dietary choices (e.g. religious dietary practices) (Young 1990; Fraser 2008). Furthermore, since the geographies of the studies were limited predominantly to North America and Europe, we found a significant gap in the literature on dairy alternatives in other regions. Evidence from other regions could, for example, provide better understanding of how dairy alternatives are perceived across different religions, geographies and cultural traditions or beliefs. Finally, there is little evidence of how supply-side actors are recognised within the dairy alternatives sector, for example social and power relations of producers and workers in PBM ingredient crop supply chains (Clay et al. 2020b). These findings are mirrored across reviews on new and emerging technologies in food and agriculture (Bunge et al. 2022; Lemarié and Marette 2022) as well as on plant-based foods (Lonkila and Kaljonen 2021), where current evidence is disproportionately consumer-oriented.

## Conclusion

This systematic scoping review aimed to contribute to the emerging field of research on social and environmental justice in food systems transitions (Huan-Niemi et al. 2020; Kaljonen et al. 2021; Tribaldos and Kortetmäki 2022) by exploring the current state of literature on dairy alternatives and recommending avenues for future research. For this review, we developed a set of research questions for assessing risks and benefits of dairy alternatives based on the criteria for just transitions (distributional, procedural and recognition); these research priorities were used to analyse the nature, scope and range of current evidence as well as highlight gaps in the literature.

The review revealed that evidence on the role of dairy alternatives in just food system transitions has mostly addressed interrelated procedural and recognitional justice concerns on the consumer and retail side of the food system, predominantly in North American and European contexts. The literature highlights how dairy alternatives, as market-driven solutions to problems in the dairy sector and its negative externalities, may be reproducing procedural injustices inherent in neoliberal governance of the food system. We find that an overreliance upon ethical consumption and responsible corporations absolve the state of responsibility for just food system transitions away from dairy, as evidenced by disproportionate government support for incumbent actors in the dairy sector (Vallone and Lambin 2023). Dairy alternatives thus exemplify the inequities of market-led food system transitions, where participation is limited to those who can afford and access certain products. This review concludes that gaps in the dairy alternatives literature addressing procedural justice regard effective communication strategies to enhance food literacy of dairy alternatives and capabilities, responsible innovation and power relations in the dairy alternative sector.

Overall, our review found that dairy alternatives present several opportunities for recognitional justice in food systems transitions, particularly in the growing visibility of multispecies welfare—despite dairy alternative marketing largely avoiding reference to animal welfare. However, gaps remain in understanding how social dimensions like class, race, and gender influence consumption, food literacy and accessibility of dairy alternatives, especially outside of North American and European contexts, and the recognition of more vulnerable supply side actors.

The review uncovered several distributional justice implications of dairy alternatives, in particular regarding affordability and nutritional deficiency risks. However, findings relating to multi-species welfare, the nutrition of more vulnerable population groups, and supply side innovation and adaptive capacities was limited. Most studies were found to adopt a liberal conceptualisation of justice as fair processes, such as carbon taxes to make PBM more affordable, without considering outcomes for other food system actors (Rawls 1971; Sen 2009; Clegg et al. 2021; Huang et al. 2022). Future research should support anticipation of distributional risks and benefits of dairy alternatives, including scenario and telecoupling analyses of transition pathways (e.g. Newman et al. 2022; Collingridge 1980; Stilgoe et al. 2013). For example, one unexplored risk identified by Clay et al. (2020b) is that the growth of dairy alternatives, facilitated by the consolidation of smaller operations by leading companies like Danone (Morris et al. 2019), could further drive large-scale dairy intensification. This risk poses implications most immediately for the capabilities of small-scale dairy operators and the welfare of dairy cows and ecosystems (Clay et al. 2020a; Shields and Orme-Evans 2015).

Dairy alternatives offer significant potential to reduce the carbon footprint of dairy consumption and improve public awareness of the environmental and animal welfare impacts of dietary choices. However, in an absence of just transition planning and coherent policy intervention, the findings of this review suggest they could also entrench inequalities and power disparities across the food system (Kaljonen et al. 2021; Huan-Niemi et al. 2020; Puupponen et al. 2017). Therefore, we recommend further empirical research to address critical questions of social and environmental justice in the dietary transition to dairy alternatives, and employ anticipatory governance principles to support more inclusive and evidence-based planning and policymaking for both just and sustainable food system transitions.

## Supplementary Information

Below is the link to the electronic supplementary material.

Supplementary file1 (DOCX 28 KB)

Supplementary file2 (XLSB 52 KB)
