# Supplementary material for: The role of dairy alternatives in just food system transitions: a scoping review
Source: Agric Human Values. 2024 Nov 14;42(2):1191–206. doi: 10.1007/s10460-024-10659-z (PMC12098427; doi:10.1007/s10460-024-10659-z)
Supplement: Supplementary file 1 — Supplementary file1 (DOCX 28 KB) [file 10460_2024_10659_MOESM1_ESM.docx]

**Supplemental Material**

Table S1: Charting table

| **Item** | **Descriptions (examples of categories)** |
| --- | --- |
| Author(s) |  |
| Title |  |
| Year of publication |  |
| Publication |  |
| Type of evidence source | Primary/secondary research |
| Region(s) |  |
| Aims/objectives of the study | Describe stated objective |
| Study population (if applicable) | By age, sex, or physical condition (if human population), consumers |
| Methodology | Quantitative, qualitative, or mixed-methods |
| Study type | Observational studies, Case studies, Content/discourse analysis |
| Methods | Detailed description of methods used |
| Disciplinary background | Business and Marketing, Geography, Law, Public Health Nutrition |
| Research focus | Brief thematic description |
| Outcomes | Describe outcomes of study |
| Primary theme | Identify which dimension(s) of justice the article relates to (see Table 2 for examples). |
| Comment on themes | Describe how it relates to primary theme, and any others |
| Main justice dimension | Category of justice the evidence falls into |

Table S2: Charting document

An Excel file supplied
